# Long-term Practice with Domain-Specific Task Constraints Influences Perceptual Skills

**DOI:** 10.3389/fpsyg.2017.01387

**Published:** 2017-08-14

**Authors:** Luca Oppici, Derek Panchuk, Fabio R. Serpiello, Damian Farrow

**Affiliations:** ^1^Institute of Sport, Exercise and Active Living, Victoria University, Melbourne VIC, Australia; ^2^Movement Science, Australian Institute of Sport, Canberra ACT, Australia

**Keywords:** attention orientation, performance, passing skill, soccer, futsal, football

## Abstract

The long-term impact of practice with different task constraints on perceptual skill is relatively un-explored. This study examined the influence of extensive practice, i.e., more than a 1000 h of structured practice, with domain-specific task constraints on perceptual skill associated with the passing action. Despite performing the same passing skill, it is not known whether long-term exposure to specific soccer or futsal task constraints influences the players’ attunement to environmental information. This study examined this issue by assessing the attention orientation of soccer (*n* = 24) and futsal players (*n* = 24) during modified games (6 vs. 6). Futsal players had higher scanning behavior during ball reception and control (40% more ball-player attention alternations) while soccer players mainly scanned the environment when not in ball possession (25% more attention alternations). We suggest that the behavioral differences found are elicited by the extensive domain-specific practice. That is, the higher number of players in soccer, and by a more intense game and easier to control ball in futsal. This study provides new insights into the long-term effects of practicing with specific task constraints.

## Introduction

The constraints-led perspective (Newell, 1986) contends that human behavior in goal-directed activities emerges as a result of the self-organization of interacting constraints (Newell, 1986; Davids et al., 2008). Constraints have been defined as boundaries or features that limit (and enable) the behavior of individuals and have been classified into three categories including: organismic, environmental and task (Newell, 1986). With practice, skilled performers develop the ability to functionally organize an optimal coordination pattern that best suits the contextual demands. Task constraints (e.g., rules, equipment, field dimension, etc.) have been the main constraint examined in sport and exercise research (Araújo et al., 2004; Davids et al., 2008) as they can be readily manipulated by practitioners to promote appropriate movement adaptations. For example, research has investigated the influence of task constraints on skill execution, showing variations in tennis performance (e.g., successful hits) and types of movement when equipment size was modified (Buszard et al., 2014a; Timmerman et al., 2015; Fitzpatrick et al., 2016), and the manipulation of task constraints to promote skill acquisition (Farrow et al., 2016), highlighting a higher number of hitting opportunities and individuals’ engagement during training with children-scaled tennis equipment (Farrow and Reid, 2010). While this research has largely focused on the physical aspects of performance, the influence of task constraints on the development of perceptual processes underpinning performance has remained relatively un-explored.

Sport expertise is characterized by superior perceptual skills that allow experts to identify and use relevant environmental information to successfully guide their actions (Williams et al., 1999; Williams and Ford, 2008). Athletes explore the vast amount of information contained in the sporting environment and learn, with practice, to exploit the information that specifies task-relevant attributes from the environment (Gibson and Pick, 2000; Savelsbergh et al., 2004). The development of expertise, thus, entails the education of attention toward information that is suited to the task at hand (Beek et al., 2003). In this context, domain-specific task constraints that specify different information and afford different types of action are expected to influence the athlete’s search for environmental information, thus, shaping the development of perceptual skills (Newell, 1991; Ericsson and Lehmann, 1996; Williams et al., 2004; Phillips et al., 2010).

Task constraints, including the type of cues athletes are required to respond to, instructions, and equipment, have been shown to affect the attentional processes underlying performance. For example, the pattern of eye movements, which assesses attention control (Panchuk et al., 2015), was different when goalkeepers responded to video simulations or to an actual opponent, with higher mean fixation locations in the *in situ* condition (Dicks et al., 2010). Visual search behaviors of youth soccer players were also influenced by the number of players, number of fixations increased as the number of players increased, and the teammate: opponent ratio contained within the stimuli presented, 3 vs. 2 condition had higher fixation order than other conditions (Vaeyens et al., 2007a). The interaction between thought processes and visual search was altered when the task required athletes to respond to a far or near stimulus in a decision-making task, higher number of fixations in the far condition and more predictive and planning statements in the near condition (Roca et al., 2013). Furthermore, equipment, i.e., standard or occluding-vision equipment, and instructions, i.e., attention focus on hole or ball marker, affected quiet eye duration and dwell time in a golf putting task (Panchuk et al., 2014). Despite providing evidence that task constraints influence perceptual skills, these studies are limited to acute changes and the long-term impact of practice with different task constraints on perceptual skills remains unclear. Here we examine the influence of extensive practice (i.e., more than a 1000 h of structured practice) with different sport-specific constraints on attention orientation underpinning the passing skill in futsal and soccer.

Futsal is the 5-a-side indoor form of football, whereas association football (also called soccer) is the 11-a-side outdoor form of football, both of which are officially regulated by the Fédération Internationale de Football Association (FIFA). The two sports share many similarities (e.g., the kicking action and the rule permitting the use of the hands other than for the goalkeeper) but various constraints differentiate the two games (see **Table 1**), and could be expected to influence the type of information players become attuned to when performing passes. In this context, several elite soccer players including Cristiano Ronaldo, Messi, and Pele stated that practicing futsal early in their career facilitated the development of decision-making skill. The smaller space, more intense man-marking, and higher opponent pressure in futsal is believed to encourage players to make and execute quicker decisions than soccer (UEFA, 2017). These experiences suggest that differences between futsal and soccer might, indeed, influence the development of perceptual skill.

**Table 1:** Soccer-specific and futsal-specific constraints with the expected influences on the game.

	Sport		
Constraint	Soccer	Futsal	Expected influence
Pitch size	100 m × 65 m	40 × 22.5	Higher game intensity and more opponent pressure in futsal
Number of players	11 vs. 11	5 vs. 5	
Individual playing area	295 m^2^/player	90 m^2^/player	

Ball: Circumference:	68.5–69.5 cm	62.5–63.5 cm	More regular and
Weight:	420–445 gr	410–430 g	predictable ball bounce in
Height of first bounce:	135–155 cm	55–65 cm	futsal

Surface	Natural or synthetic grass	Flat, typically hardwood or laminated synthetic material	

Rules	Offside 3 substitutions	No offside Flying substitutions	

Despite the differences in rules and equipment, passing is the main skill performed in both sports (Rampinini et al., 2009; Mohammed et al., 2014). The passing action is complex as it involves the interception of the ball while making a decision, usually under pressure, about which teammate to pass the ball to. Players are required to balance their attention between information specifying the approaching ball and the behavior of players around them. While no studies have been conducted in futsal, separate studies have investigated how visual attention underpins decision making and ball control in soccer. The analysis of eye movements is frequently used as proxy for examining attentional processes due to partial-interdependence of attention and eye movements (Van Gompel et al., 2007). While attention can move independent of the eyes, gaze shifts are strictly correlated with shifts in attention (Henderson, 2003) and gaze patterns provide information on individuals’ control of attention (Panchuk et al., 2015). A video-based task showed that high visual scanning behaviors (i.e., attention frequently shifting between different locations in the display) underpinned successful decision making (Vaeyens et al., 2007b), and visual information of ball behavior and foot-ball interaction supported accurate performance in a ball control task (Williams et al., 2002). Correa et al. (2014) suggested that futsal players should become attuned to information of their teammates’ behavior early in the execution of the pass; however, they did not examine the attentional processes underpinning such behavior. As such, it is not known how soccer and futsal players control their attention during games, when they need to couple ball control and decision making, because *in situ* assessments of attentional strategies, which are required to accurately capture players’ behavior (Dicks et al., 2010) are rarely achieved in sport due to limitations in assessment methods. Even though the skill is the same, it is unclear how extended exposure to the different task constraints in futsal and soccer influence perceptual skills underpinning passes.

The purpose of the present study was to investigate how perceptual skill underpinning the passing action is influenced by domain-specific practice with different task constraints. The orientation of visual attention when controlling and passing the ball was examined in young, elite futsal and soccer players during modified games. We hypothesized that, despite performing a similar skill, sport-specific constraints would promote attunement to different information during the execution of the pass. Specifically, the higher game intensity (i.e., higher opponent pressure and quicker passes) and ball characteristics (i.e., more predictable and easier to control) of futsal are expected to promote an orientation of the futsal players’ attention toward the behavior of the players around them and to force quick decisions, relative to soccer players who would mainly orient their attention toward the ball. Furthermore, the players’ orientation of attention was also assessed when their team was in possession of the ball. The higher number of players on the pitch in soccer was predicted to encourage soccer players to orient their attention toward teammates’ and opponents’ movement for a greater period of time than futsal players.

## Materials and Methods

### Participants

A total of 48 elite, young, male players were recruited for the experiment. They were divided into two groups based on their domain of expertise: soccer players (*n* = 24, 13.6 ± 1.2 years old, 6.8 ± 1.2 years of experience) and futsal players (*n* = 24, 13.6 ± 1.2 years old, 7.0 ± 1.6 years of experience). Participants completed a questionnaire on their training history that included the number of training years at club level, and an average amount of training months per season, number of training per week and training duration. The years of experience refer to structured team practice in their domain, at an elite level. Players in both groups, on average, had three 90 min sessions per week, 40 weeks per year, resulting in approximately 1220 and 1260 h of domain-specific structured practice in the soccer and futsal group, respectively. Furthermore, both groups had participated in approximately 400 competitive games in their respective domain. To control for training and competition time, two squads (U13 and U15) from within one of the most successful Spanish teams and the most successful Australian State representative squads, respectively, were recruited. Both groups had a similar weekly training schedule that included four 90 min training sessions and one competitive game. Only outfield players were sampled from the squads. Players that trained at a club level in the ‘other’ sport were excluded. As such, participants had experience only in their own sport. Technical issues during data collection (e.g., the scene camera moved during the game, the video quality was unreliable, etc.) resulted in a final sample size of 17 futsal players (U13 = 9 and U15 = 8) and 20 soccer players (U13 = 9 and U15 = 11) being examined.

Prior to the study, participants were fully informed of the risks involved in participating in the experiment and their parents or guardian gave written consent for them to participate. The study was approved by the research team’s University Ethics Committee.

### Experimental Task

**FIGURE 1:**
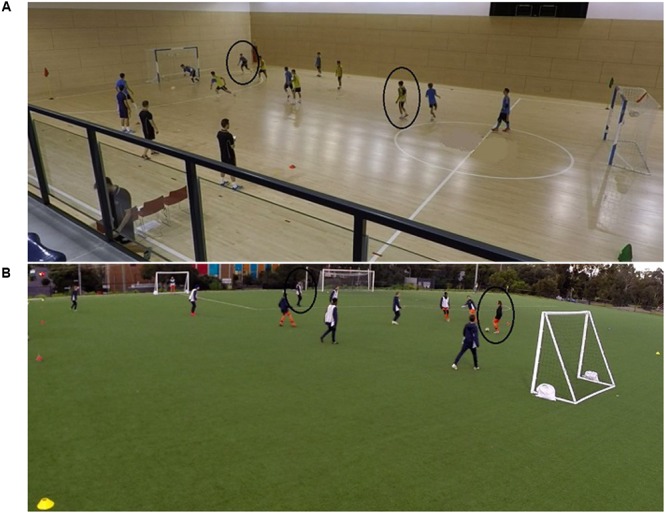
Example of futsal task (FUT) task **(A)** and soccer task (SOC) task **(B)**. The two participants wearing the scene camera are highlighted in the circles. The goals were futsal-size goals to discourage shooting and encourage passing in the SOC task.

The experimental task was a 5 vs. 5 (plus goalkeeper) modified game (**Figure 1**). The game format, the aim (scoring goals to win), and the rules, including no offside rule, were the same for both sports to allow for between-sport comparisons. However, the ball, playing surface, and individual playing area (IPA) were manipulated to create the two domain-specific tasks. As such, the soccer task (SOC) was performed with a FIFA quality-approved soccer ball, on a synthetic-grass pitch of 24 m × 36 m corresponding to an IPA of 86 m^2^/player, which is representative of an actual soccer game (Fradua et al., 2013). The futsal task (FUT) was performed with a FIFA quality-approved futsal ball, on a wooden pitch of 24 m × 15 m corresponding to an IPA of 36 m^2^/player, which is the most common density in futsal matches (unpublished observations). The two tasks were not simulations of soccer and futsal games but representative of the passing skill in the two disciplines.

The scene camera of a mobile eye tracking system (Mobile Eye, Applied Sciences Laboratories, Bedford, MA, United States) was used to collect participants’ attention orientation, consistent with Pluijms et al. (2016) previously validated methodology. An external camera (GoPro 3+) was placed in one corner of the pitch to record the task.

### Procedure

All familiarization and testing sessions were performed in the participant’s regular training environment (i.e., futsal group in FUT and soccer group in SOC). The groups performed a familiarization session which consisted of a shortened version of the experimental task 1 week prior to the experimental session. The experimental session comprised of six games that were 5 min in duration with 5 min breaks between games to allow players time to recover. Two participants wore the scene camera during each game and the participants rotated between tasks (e.g., participants A and B wore the camera in game 1, then participants C and D wore the camera in game 2, and so on) to assess all 12 players throughout the session. As a result, each player wore the scene camera during one game and participated in five games in total, having rested the game prior to wearing the scene camera. Players were instructed to perform the task as they normally would; aiming to win each game, with the only added rule of no slide tackles to the player wearing the eye tracker for safety reasons.

After a standardized, 10 min warm-up, each group was randomly divided into two teams of six players including a goalkeeper. Before each task, two participants were fitted with the Mobile Eye. The task started with the investigator bouncing the ball on the ground. The bounce was used as reference to synchronize the eye-tracker scene camera and the external camera. One of the investigators umpired the games.

### Data Analysis

#### Attention Orientation

The center of the image from the scene camera was used as a reference to classify attention orientation into two areas of interest, consistent with Pluijms et al. (2016). The orientation of attention was classified either as ball-directed, whenever the ball was in the center of the image, or as player-directed, when the ball was not in the center. These two areas are relevant during the passing action as players are required to direct their attention toward other players’ behavior, to inform decision making, and toward the ball, to guide skill execution. Furthermore, players can direct their attention toward either the player in possession of the ball or other areas, when not in possession of the ball. Previous studies have created a specific variable, fixation order, to evaluate the players’ ability to alternate their attention between ball-related and player-related areas (Williams et al., 1994; Williams and Davids, 1998). Research showed that the ability to frequently alternate attention between these two areas underlies successful decision making (Vaeyens et al., 2007b) and discriminates elite to sub-elite young soccer players (Vaeyens et al., 2007a). Furthermore, the evaluation of ‘exploratory’ visual behavior of English Premier League players, assessed through video analysis, showed a positive relationship between ball-player gaze alternations and passing performance (Jordet et al., 2013).

The footage from the scene camera and the external camera, both recorded at 30 hz, were synchronized using Sports Code to couple the orientation of attention with specific events, consistent with the Vision-in-Action procedure (Vickers, 1996).

The game was divided into three phases, reception, control, and team phase. The *reception phase* referred to the period from the time the ball left a teammate’s foot to the participant’s first touch. The *control phase* captured the time in which participant had possession of the ball, from the first touch to the release of the pass. The *team phase* referred to the overall time the participant’s team had possession of the ball minus the previous two phases. Three attention-orientation variables were determined: relative attention-orientation time (AT), attention-orientation switches (AS), and last-attention location (AL). AT and AS were adapted from previous research on visual search strategies (Williams et al., 1994; Williams and Davids, 1998), representing relative viewing time and fixation order, respectively. AT refers to the relative percentage of time the attention was oriented on the two areas of interest during the passing action. AS was calculated as the number of times participants alternated the attention between the two areas of interest per second. AL was specifically designed to capture the participants’ behavior during the passing action and refers to the location where the head was oriented when the foot made contact with the ball, when controlling the ball (reception phase) or when performing the pass (control phase). These dependent variables were evaluated frame-by-frame using Sports Code. AT and AS were evaluated, separately, in each phase, whereas AL was examined only in the reception and control phases.

Passes where the receiving pass (from the teammate) was bouncing and the participant’s body was oriented toward the side line (with less than four players in front of him) were excluded from the analysis to limit the influence of external factors. Furthermore, only passes that included the control phase were considered (i.e., direct passes were excluded). This resulted in a total of 350 passes analyzed, 169 in futsal (10 ± 4 passes per player) and 181 in soccer (9 ± 2 passes per player).

#### Game Dynamics

Three game-related variables were assessed to quantify the context in which the passes were performed. Technical intensity of the game referred to the number of passes performed each minute during the game. IPA is a tactical variable that can influence a player’s decision (Fradua et al., 2013) and is the playing area the player was in when performing the pass. To calculate IPA, the pitch was divided into fixed squares of known dimensions (i.e., 108 m^2^ in SOC task and 60 m^2^ in FUT task), using side-line cones as references, and the number of players inside the square the participant was occupying when performing the pass were counted. The dimension of the square was then divided by the number of players to get the IPA (e.g., in SOC task, 108 m^2^/5 players = 21.5 m^2^/player). Reception time referred to the time it took the ball to travel from the teammate to the participant with the eye tracker (i.e., reception-phase time). This indicates how much time the participants had to prepare the reception and control action.

### Coding Reliability

Five percent of the total trials were randomly selected for inter- and intra-coder reliability. The trials were recorded and coded by the primary coder a week after the original coding was completed, and by a second coder. Cohen’s kappa was 0.94 for intra-coder reliability and 0.93 for inter-coder reliability, all representing perfect agreement (Landis and Koch, 1977).

### Statistical Analysis

All the attention-orientation dependent variables were analyzed separately using generalized linear mixed modeling (Proc Glimmix in Version 3.6 of Statistical Analysis System Studio, SAS Institute, Cary, NC, United States) with domain (soccer, futsal) as a fixed factor and participants as a random factor. Allowance was made for overdispersion. Specifically, Poisson regression was computed to analyze AS as data was expressed as count per unit of time (switches/sec), whereas Logistic regression was computed for AT and AL, being both binary dependent variables. The between-subject standard deviation for the standardization of the effect sizes was calculated using the pure observed between-subject variance and the overdispersed sampling variance. The game dynamics variables were analyzed separately using an independent *t*-test.

Significance was set at *p* < 0.05 for all the analyses and the magnitude of changes was assessed using Effect Sizes (ES) with 90% Confidence Intervals defined as follows: <0.2 trivial, 0.2–0.6 small, 0.6–1.2 moderate, 1.2–2.0 large, >2.0 very large (Hopkins, 2010).

## Results

### Attention-Orientation Data

The mean data for attention-orientation variables are presented in **Table 2**.

**Table 2:** Mean relative attention-orientation time on player-directed area (AT, in percentage), attention-orientation switches (AS, in switches/sec) and last-attention orientation on ball-directed area (AL, in percentage) ± SD of the two groups across the three different phases.

		Group		
Phase	Attention-orientation	Futsal	Soccer	*p*-Value
Reception	AT (%)	17 ± 12	12 ± 8.0	*P* = 0.59
	AS (switches/sec)	1.0 ± 0.5	0.6 ± 0.3	*P* = 0.08^∗^
	AL (%)	54 ± 21	16 ± 17	*P* < 0.01^∗∗∗∗^
Control	AT (%)	45 ± 18	32 ± 18	*P* = 0.01^∗∗^
	AS (switches/sec)	1.1 ± 0.4	1.1 ± 0.4	*P* = 0.30^∗^
	AL (%)	20 ± 23	14 ± 21	*P* = 0.43^∗^
Team	AT (%)	13 ± 5.0	22 ± 10	*P* < 0.01^∗∗^
	AS (switches/sec)	0.3 ± 0.1	0.4 ± 0.1	*P* = 0.01^∗∗^

*Effect size: small^∗^, moderate^∗∗^, large^∗∗∗^, very large^∗∗∗∗^.*

#### Reception Phase

There was a significant group difference in AL (*p* < 0.01, ES = 2.03 ± 0.6). Prior to their first touch, futsal players oriented their attention primarily toward other players relative to the soccer players (54% of the time vs. 16% of the time). The results also showed a small, near significant effect in AS (*p* = 0.08, ES = 0.22 ± 0.21) with futsal players switching their attention between ball and players more frequently than soccer players. No differences were found in AT (*p* = 0.59, ES = 0.18 ± 0.56).

#### Control Phase

There was a significant group difference in AT (*p* = 0.01, ES = 0.86 ± 0.58). Futsal players oriented their attention toward other players for a longer period of time than soccer players (45% vs. 32%). No differences were found in AS (*p* = 0.30, ES = 0.30 ± 0.49) and AL (*p* = 0.43, ES = 0.27 ± 0.57).

#### Team Phase

There was a significant group difference in AT (*p* < 0.01, ES = 1.02 ± 0.57). Soccer players oriented their attention toward other players longer than futsal players (22% vs. 13%). There was also a significant group difference in AS (*p* = 0.01, ES = 0.85 ± 0.57). Soccer players alternated their attention more frequently between ball and players than futsal players.

### Game Dynamics Data

The mean data for game dynamics variables are presented in **Table 3**. There were significant group differences in reception time (*p* < 0.01, ES = 1.69 ± 0.55), in IPA (*p* < 0.01, ES = 2.99 ± 0.60), and in technical intensity (*p* < 0.01, ES = 6.21 ± 0.53). The results showed a higher game intensity in futsal with more passes per minute performed (35%), shorter time to organize the controlling action (30%) and lower IPA (46%) relative to soccer.

**Table 3:** Mean technical intensity (number of passes/min), individual playing area (IPA, m^2^/player) and reception time (seconds) ± SD of the two groups.

	Group		
Game dynamics	Futsal	Soccer	*p*-Value
Technical intensity	30.9 ± 2.1	20.1 ± 1.3	*P* < 0.01^∗∗∗∗^
IPA	24.9 ± 4.9	46.5 ± 11.9	*P* < 0.01^∗∗∗∗^
Reception time	0.83 ± 0.13	1.08 ± 0.17	*P* < 0.01^∗∗∗^

*Effect size: small^∗^, moderate^∗∗^, large^∗∗∗^, very large^∗∗∗∗^.*

## Discussion

The aim of this study was to investigate how extensive practice with domain-specific task constraints influenced perceptual skills associated with the passing action. We hypothesized that futsal-specific constraints (e.g., high game intensity, ball characteristics, playing surface, etc.) would promote an orientation of attention toward player-directed areas while soccer constraints would encourage a ball-directed orientation of attention. The results confirmed our hypothesis as futsal players spent more time orienting their attention toward player-directed areas and alternated their attention more frequently between the ball and players during the reception and control phases. Futsal players developed perceptual-motor coordination that allowed them to perform the first touch while orienting their attention toward player-directed areas, and then spent more time attending to players during ball control. On the other hand, soccer players alternated their attention between the ball and players less frequently, and mainly attended to the ball when performing the first touch. Furthermore, they spent less time orienting their attention toward players during the control phase. Our second hypothesis was that the higher number of players on the pitch in soccer would promote a higher frequency scanning behavior in soccer players during the team phase. This prediction was confirmed as soccer players spent more time orienting their attention toward players and alternated their attention more frequently between the ball and other players than futsal players during the team phase. These findings demonstrate that each group developed unique perceptual strategies to gather information about the ball and other players at different phases during the games. Furthermore, the smaller individual-playing area, shorter reception time and higher technical intensity in futsal, highlighted, as predicted, an overall higher game intensity in futsal.

While our methodology did not allow us to evaluate the influence of each sport-specific constraint on the observed behaviors, we can speculate how the constraints may have influenced the development process. The higher intensity of the futsal game, confirmed in this study (i.e., smaller IPA, shorter reception time and technical intensity), is suggested to be the main constraint that led futsal players to scan the environment and focus their attention on player-directed areas just prior to and during ball control. Similarly, it has been suggested that futsal players should detect information earlier during the passing action to make decisions (Correa et al., 2014). Furthermore, we speculate that the futsal ball, which has a predictable bounce and is easier to handle, contributed to the development of the observed behavior during the futsal players’ first touch. On the other hand, the higher number of players in soccer (i.e., more potential information sources) and the less predictable behavior of the soccer ball could be the key constraints that led soccer players to scan the environment more frequently when their teammates were in possession of the ball and to orient their attention to the ball when they are controlling it. Previous research has shown that a higher number of players within a players field of view promoted a higher number of visual fixations (Vaeyens et al., 2007a). Furthermore, stimuli that are close to the player, i.e., ball and other players, promote lower visual search rates than stimuli that are far from the player (Roca et al., 2013). In the team phase, the proximity of stimuli in the two domains could play a role in shaping behavior as futsal players are accustomed to dealing with stimuli that are closer to them, than soccer players due to the smaller size of the futsal pitch. A potential research direction would be to investigate the influence of these constraints independently.

The last-attention orientation (AL) in futsal players was mainly player-directed in the reception phase indicating that they developed a coordination pattern that allowed them to receive the ball while orienting their attention toward other players, while soccer players’ attention was mainly ball-directed. This suggested that futsal players had automated control of the ball as they oriented their attention away from the ball in the last phase of ball flight, which is the moment that requires the highest attentional demands in interceptive actions (Williams et al., 1999). Previous research has shown that modified equipment that simplifies skill performance (e.g., scaled tennis equipment for children) promotes implicit processes that eventually lead to movement automaticity (Buszard et al., 2014b). We suggest that, in this study, the futsal ball and the hard futsal-court surface are likely to be the constraints that simplify ball control and delivery, potentially promoting more enhanced movement automaticity.

This study is novel, having assessed attentional processes *in situ*, coupling perception and action, in two team sports. Laboratory-based tasks typically do not include the reception of the ball when examining decision making in team sports. Participants usually watch patterns of play and are required to perform sport-specific action using a ball positioned at their feet (Helsen and Starkes, 1999; Vaeyens et al., 2007b) or in their hand (Gorman and Farrow, 2009; Furley et al., 2010). Interestingly, one of the key findings of our study was the pattern of perceptual-motor coordination developed by futsal players during the reception phase (i.e., AL on player-directed area). This difference in coordination would not have been captured if we had not designed a task in which players had to receive and pass the ball. This suggests future studies need to consider the inclusion of a ball reception phase when examining decision making in team sport, as it might be critical for players’ coupling of perception and action.

This study provides new evidence on the influence of task constraints on perceptual-motor skills. Previous research has been limited to acute changes (Dicks et al., 2010; Panchuk et al., 2014; Timmerman et al., 2015), whereas this experiment examined the long-term effects of practicing with specific task constraints. Players developed patterns of coordination that best suited the different contextual demands, e.g., higher game intensity, different ball, etc. This supports the notion that individuals’ coordination was the result of self-organization of interacting constraints, particularly task constraints in the current study (Newell, 1986; Davids et al., 2008). It also highlights the importance of manipulating constraints on action to better understand human coordination and skill acquisition (Newell, 1986).

The findings of this study also have practical implications for sport practitioners. Researchers have suggested manipulating task constraints, including equipment and rules, to obtain desired changes and adaptations in athletes’ behavior as it allows individuals to find their own preferred pattern of coordination (Araújo et al., 2004; Davids et al., 2008; Farrow et al., 2016). In this context, our study suggests that the type of ball, the number of players and the IPA can be manipulated to promote different perceptual skills. The futsal ball could potentially be used on a hard surface to develop automaticity of the kicking skill. Similarly, previous research has showed the benefit of using a futsal ball to acquire ball juggling skill (Button et al., 2005). Furthermore, the IPA and number of players can be manipulated to constrain the players’ visual search for environmental information. The number of players can be increased to promote higher scanning behavior as indicated by more frequent attention switches and more time spent on players in the soccer group. IPA can be manipulated to modify opponent pressure when performing passes, which in turn, encourage players to search for decision-making information at key moments. For example, high opponent pressure can be used to promote early and quick detection of environmental information.

We interpreted players’ behaviors observed in this study as being the result of long-term practice with different task constraints. However, we acknowledge that other interpretations cannot be excluded. For example, players might have developed different motor programs in response to the different domain-specific training that have influenced the behavior. Furthermore, factors other than task constraints might have contributed to the development of the behaviors. Given that we only captured participant’s performance during a single session we were not able to determine how these skills developed over time. It also possible that only the players that developed the observed behaviors early in practice continued to participate in the sport (i.e., players self-selected to participate in either sport based on these behaviors). These limitations should be addressed in future research to further examine how different task-constraints influence the development of perceptual skill. Longitudinal studies, with baseline assessment, training and post-training assessment are needed to examine the causal relationship. Furthermore, attention orientation was assessed on a single occasion in this study, and future research could assess the behavior over multiple occasions to evaluate the adaptability of the skill.

In summary, despite performing the same skill (passing action), futsal and soccer task constraints shaped athletes’ perceptual skills. Higher game intensity, higher opponent pressure, an easier-to-handle ball, and a lower number of players in futsal led futsal players to acquire information on other players’ behavior just prior to and during ball control. On the other hand, a higher number of players, lower game intensity and an unpredictable ball behavior in soccer led soccer players to scan the environment when not in possession of the ball. Despite the limitations highlighted in the previous paragraph, this is the first study showing the influence of task constraints on perceptual skills in sport and a potential future research direction would be to examine the influence of task constraints independently in a longitudinal study.

## Author Contributions

LO, DP, FS, and DF substantially contributed to the design of the work and the interpretation of the data, LO acquired and analyzed the data. LO drafted the work, LO, DP, FS, and DF revised the work critically. LO, DP, FS, and DF approved the final version to be published. LO, DP, FS, and DF agreed to be accountable for all aspects of the work.

## Conflict of Interest Statement

The authors declare that the research was conducted in the absence of any commercial or financial relationships that could be construed as a potential conflict of interest.
